# Intrauterine IPEX

**DOI:** 10.3389/fped.2020.599283

**Published:** 2020-11-20

**Authors:** Magda Carneiro-Sampaio, Carlos Alberto Moreira-Filho, Silvia Yumi Bando, Jocelyne Demengeot, Antonio Coutinho

**Affiliations:** ^1^Laboratory of Medical Investigation (LIM-36, HCFMUSP), Department of Pediatrics, Faculdade de Medicina da Universidade de São Paulo, São Paulo, Brazil; ^2^Instituto Gulbenkian de Ciência, Oeiras, Portugal

**Keywords:** IPEX, immune fetal hydrops, intrauterine fetal deaths, IPEX-like syndromes, IL2RB, neonatal-onset autoimmune diabetes, fetal ultrasonography, recurrent male miscarriages

## Abstract

IPEX is one of the few Inborn Errors of Immunity that may manifest in the fetal period, and its intrauterine forms certainly represent the earliest human autoimmune diseases. Here, we review the clinical, histopathologic, and genetic findings from 21 individuals in 11 unrelated families, with nine different mutations, described as cases of intrauterine IPEX. Recurrent male fetal death (multigenerational in five families) due to hydrops in the midsemester of pregnancy was the commonest presentation (13/21). Noteworthy, in the affected families, there were only fetal- or perinatal-onset cases, with no affected individuals presenting milder forms with later-life manifestation. Most alive births were preterm (5/6). Skin desquamation and intrauterine growth restriction were observed in part of the cases. Fetal ultrasonography showed hyperechoic bowel or dilated bowel loops in the five cases with available imaging data. Histopathology showed multi-visceral infiltrates with T lymphocytes and other cells, including eosinophils, the pancreas being affected in most of the cases (11/21) and as early as at 18 weeks of gestational age. Regarding the nine *FOXP3* mutations found in these cases, six determine protein truncation and three predictably impair protein function. Having found distinct presentations for the same *FOXP3* mutation in different families, we resorted to the mouse system and showed that the scurfy mutation also shows divergent severity of phenotype and age of death in C57BL/6 and BALB/c backgrounds. We also reviewed age-of-onset data from other monogenic Tregopathies leading to IPEX-like phenotypes. In monogenic IPEX-like syndromes, the intrauterine onset was only observed in two kindreds with *IL2RB* mutations, with two stillbirths and two premature neonates who did not survive. In conclusion, intrauterine IPEX cases seem to constitute a particular IPEX subgroup, certainly with the most severe clinical presentation, although no strict mutation-phenotype correlations could be drawn for these cases.

## Introduction

IPEX (Immunodysregulation Polyendocrinopathy Enteropathy X-linked syndrome) is one of the few Inborn Errors of Immunity whose manifestations can appear as early as fetal life, and its intrauterine forms certainly represent the earliest autoimmune diseases in humans. Since it was first reported, two decades ago, that inactivating *FOXP3* mutations cause IPEX (1, 2), it was clear that its clinical features usually manifest early in life and, in some cases, are already present at birth. Among the 94 reported IPEX cases with “age-of-onset” information, 46 (49%) presented the first symptoms during the neonatal period, and 11 presented typical features at birth or in the first 2 days of life (11.5%), drawing attention to antenatal initiation of the pathological process (3).

However, only in 2014, IPEX *in utero* was definitely identified by Xavier-da-Silva et al. (4). These authors described two unrelated families with *FOXP3* mutations. One of these families had a history of recurrent multigenerational male miscarriages, and a new pathogenic variant (c.319_320delTC) was detected in monochorionic twins with hydrops at 21 weeks of gestational age. The other family had an equivalent history of intrauterine fetal deaths (IUFD) and a premature baby diagnosed with insulin-dependent diabetes mellitus (IDDM) in the 1st hours of life, thus confirming that IPEX may manifest *in utero*. The patient, his mother, and maternal grandmother had the *FOXP3* mutation (c.1189C > T), which has been previously detected only in another family with all babies presenting manifestations at birth (5). Several other equivalent observations were reported in the following years (Table 1). Here, we focused on the clinical, histopathological, imaging, and genetic characterization of the 11 unrelated families described in the literature with confirmed intrauterine IPEX. The age-of-onset of clinical manifestations was reviewed for most of the reported cases of monogenic IPEX-like syndromes.

**Table 1:** Main clinical, imaging, and histopathologic features of 21 individuals from 11 unrelated families with intrauterine IPEX.

**Description**	**Individual description**																				
***FOXP3*** **mutation**																					
Exon	1		3				6	7^*^	8	9					9			10^*^	11		
Codon	c.151C>T		c.319_320delTC				c.727delG	c.749delA	c.906delT	c.1009 C>T					c.1033C>T			c.1087ª>G	c.1189C>T		
Protein	p.R51X		p. S107Nfs*98				p.E243Sfs*11	p.K250Rfs*4	p. D303fs*87	p. R337X					p.L345F			p.I363V	p.R397W		
Type	Nonsense		Frameshift				Frameshift	Frameshift	Frameshift	Nonsense					Missense			Missense	Missense		
**Familial/patient history**																					
Family ID^**^	11		2^M^				7	6^M^	10^M^	4			5^M^		3			8	1^M^		9
No. of born-alive individuals			2				1	1										1	1		1
Prematurity			Y	Y			Y	Y										N	Y		Y
Time of survival			51d	2h			2y^A^	SB										29h	8m		19d
No. of male miscarriages	2				2^B^				1	3			2		3					1	
Gestational age (weeks)	20	21	31	31	21	21	35	27	18	18	20	18	18	22	24	NA	NA	39	36	27	33
**Fetal ultrasonography obs**.																					
Echogenic bowel	X	X					X	X							X						
Cutaneous lesions							X	X										X			
Hydrops	X	X			X	X		X	X	X	X	X	X	X	X					X	
IUGR							X								X				X		
Polyhydramnios							X											X			
**Presentation at birth**																					
Diarrhea			X^U^				X^L^												X		
IDDM							X^L^												X		
Cutaneous lesions								X											X		
**Necropsy findings**																					
Hydrops		X												X							
Cutaneous lesions								X	X						X						
Dysmorphic features		X						X						X							
Lymphocytic infiltrate in pancreas		X			X	X			X		X	X			X	X	X	X			X
**DNA sequencing**	X	X			X	X	X	X	X		X	X		X		X	X	X	X		X
**References**	(6)		(4)				(7)	(7)	(8)	(9)			(10)		(11)			(12)	(4)		(12)

* Mutation also identified in child or adult with IPEX;

** Affected families were listed according to chronological order of the publication;

M Multigenerational male miscarriages;

A the patient was 2 years old at time of publication (7);

B *monochorionic twins; X^L^, After 24 h; X^U^, Untreatable diarrhea; Time of survival: SB, h, d, m, and y refer, respectively, to stillbirth, hours, days, months, and years; IUGR, intrauterine growth restriction; IDDM, insulin dependent diabetes mellitus; NA, information not available; Y, yes; N, No*.

## Clinical, Imaging, and Histopathologic Features of Intrauterine IPEX

**Figure 1:**
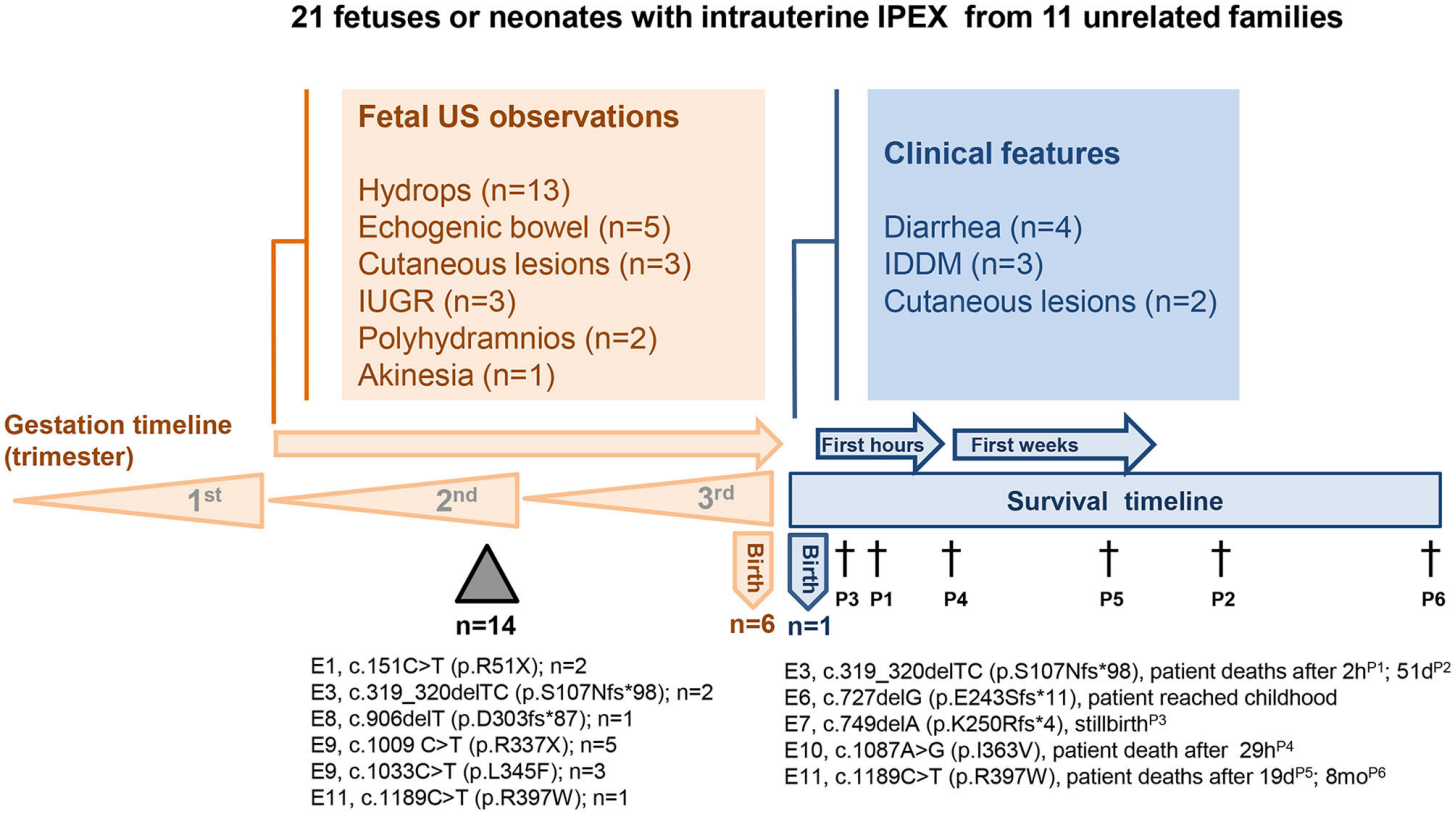
Clinical, imaging, and molecular findings in 21 fetuses or neonates with intrauterine IPEX from 11 unrelated families. *FOXP3* mutation site, protein alteration, and the number of the cases were indicated below the timelines. US-ultrasonography; E-exon; Triangle-miscarriages; †-death.

The main features of 21 affected individuals with available clinical and/or histopathological data from 11 unrelated families with nine different *FOXP3* gene mutations are summarized in Table 1 and Figure 1. Clinical, imaging, and histopathologic findings in intrauterine IPEX cases are listed in Supplementary Table 1. Family history of recurrent male miscarriages and/or neonatal male deaths was identified in nine of these 11 families, being multigenerational in five of them. Interestingly, all these families only presented intrauterine- or perinatal-onset cases, without milder cases appearing later in life (4, 7, 8, 10). The history of the family described by Shehab et al. (8) is remarkable: 19 male IUFD at 20 weeks of gestational age or less in four consecutive generations were reported, and a rare *FOXP3* frameshift mutation was identified through the whole genome sequencing analysis in five healthy obligatory carrier females and in the most recent IUFD case. One of the families that we studied (family 2 in Table 1) also had several miscarriages and neonatal male deaths in three consecutive generations. The pedigree analysis of these two large families shows an X-linked pattern of inheritance of male IUFD cases, thus reinforcing the observation that *FOXP3* mutation carriers present good health conditions (4, 8).

Most intrauterine deaths occurred in the second trimester of pregnancy due to hydrops fetalis, whose pathophysiology in these cases has not been completely elucidated yet, with many studies linking hydrops to hemolytic anemia (4, 6, 8, 10). Increased extramedullary hematopoiesis in multiple visceral organs, liver hemosiderosis, as well as increased circulating immature blood cells in placental vasculature were observed in IPEX fetuses with hydrops (4, 6, 10). There are a few observations of mild myocardium lesions that could not explain a heart failure (4, 9).

There are also descriptions of premature births in the families here analyzed, sometimes with restriction of intrauterine growth. There are families with both IUFD and preterm neonates. Mortality rate was very high among these individuals, only two of them having reached the conditions to be submitted to hematopoietic stem cell transplantation (4, 7), of which only a 35-week-gestation premature survived, incidentally the only one alive at the time of publication [Family 6, (7)], among the 21 individuals with intrauterine IPEX here analyzed (Table 1 and Figure 1).

It is noteworthy that the affected premature babies presented typical IPEX features in the 1st hours of life, i.e., insulin-dependent diabetes mellitus (IDDM) or refractory diarrhea (4, 7). Five cases presented skin manifestations, two of them with severe exfoliative erythroderma evocative of Omenn's syndrome (OS), with epidermal necrosis and dermal infiltration of lymphocytes, histiocytes, eosinophils, and giant multinucleated cells (7, 11). There was no parental consanguinity and no mutations were identified in *RAG1* and *RAG2* genes in the 24-week-gestation stillbirth with severe skin manifestations reported by Vasiljevic et al. (11), thus discarding the diagnosis of OS, a rare Inborn Error of Immunity associated with severe immunodysregulation, already described at birth in a few cases (13).

Regarding unusual manifestations, there were three fetuses with dysmorphic features (clubfeet, clinodactyly, equinovarus, and micrognathia) among the individuals here analyzed, but they also presented pathogenic variants of other autosomal genes (7, 10). Dysmorphisms and malformations have not been described in the IPEX babies (3, 14). Akinesia was another rare manifestation described in one fetus at 18 weeks of gestational age, whose necropsy showed a near absent skeletal muscle, that was replaced by fibrosis tissue and macrophages (9). T-cell myocardium infiltrate was also observed in this fetus. Moreover, in the same family, another fetus who died due to hydrops at 20 weeks also presented skeletal muscle almost replaced by fibrotic tissue and macrophages (9). Recently, fetal hydrops has been associated to akinesia (15). Another uncommon feature among individuals with intrauterine IPEX was pulmonary hypoplasia, found in only one term newborn that died from respiratory failure 29 h after birth (12).

Prenatal ultrasonography exploration revealed bowel anomalies in all individuals, except one, whose analyses were described: hyperechoic bowel, prominent fluid-filled loops of bowel, and echogenic debris in the stomach being the most frequent findings (6, 7, 11, 12). Griswold et al. (16) observed dilated loops of bowel in two IPEX fetuses from the same family, who had manifestations in the neonatal period. In cases with severe dermatitis, hyperechoic skin with projections was described (7, 12).

In addition to the typical hydrops fetalis features (generalized subcutaneous edema, ascites, pleural and pericardial effusions, diffuse villous edema in placenta), necropsy of several miscarried individuals demonstrated histologic alterations in multiple organs. T-lymphocyte (CD3^+^, CD4^+^, and CD8^+^ cells) infiltrates in pancreatic parenchyma was described in many affected individuals (4, 6, 8, 9). Allenspach et al. (12) examined two unrelated IPEX newborns that died shortly after birth and described tertiary lymphoid structures in pancreas, with extensive T-cell (CD3^+^) zones, including mixed CD4^+^ and CD8^+^ cells, surrounding distinct B-cell (CD20^+^) aggregates, all cells having the XY karyoptype, besides chronic inflammatory changes and areas of fibrosis. Interestingly, in these and other cases, islets were structurally intact, thus, characterizing an exocrine-dominant pancreatitis in early life (4, 12). Allenspach et al. (12) also examined the lymphocyte repertoire and showed that TCR (T-cell receptor) compartment presented significant oligoclonal enrichment in the pancreas, compared to blood, which may indicate antigen specificity and organ-specific autoimmunity.

Polymorphous inflammatory infiltrates in pancreas, digestive tract, liver, kidneys, skin, and bronchi with T lymphocytes, histiocytes, neutrophils, eosinophils, and Charcot-Leyden crystals, a product of eosinophil breakdown, were described by Vasiljevic et al. (11) in a 24-week-gestation stillbirth with erythroderma. In a 27-week-gestation premature also with exfoliative erythroderma described by Louie et al. (7), dermal infiltration of lymphocytes, histiocytes, eosinophils, and multinucleated giant cells was observed, besides epidermal necrosis. Marked multi-organ infiltration of CD3+ lymphocytes was described involving the liver, kidneys, testis, adrenal, and thyroid glands (6, 8, 9, 12). In two unrelated cases, the spleen was described as presenting white pulp depletion (4) and reduction of lymphoid aggregates (6). Thymus histology was evaluated in only one case and the organ was described as having a normal architecture and cellularity (4). Moreover, thymic hypoplasia was found in the case described by Shanes et al. (6).

While we detail here only the individuals reported as presenting *in utero* IPEX manifestations, the literature describes other highly suggestive cases of intrauterine-onset, particularly of low-birth-weight neonates. Smith et al. (17) described a 37-week small-for-gestational age neonate with a new *FOXP3* mutation (c.1227_1235delTGAGCTGGA), who developed IDDM on the 2nd day of life, and refractory diarrhea on day 13th, and did not survive. The two siblings described by Griswold et al. (16), reported because of their ultrasonographic bowel features, presented IPEX manifestations in the 1st days of life and had a confirmed *FOXP3* mutation (c.1150A > G). The family described by Levy-Lahad and Wildin (5) had three siblings with severe intrauterine growth restriction, neonatal IDDM, and enteropathy, none of them surviving. This family had the mutation (c.1189C > T), which was described in only two other kindreds, both with intrauterine IPEX onset (families 1 and 9 in Table 1). As an overall estimate, at least in 10% of IPEX cases the disease process began in the intrauterine period.

## *FOXP3* and IPEX

**Figure 2:**
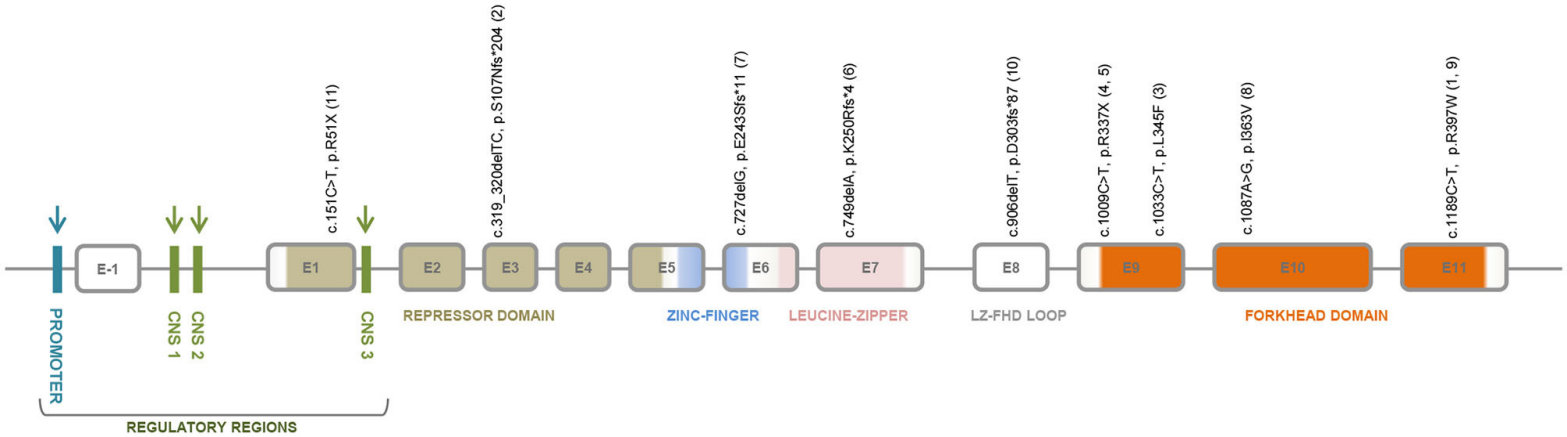
FOXP3 gene structure showing the regulatory regions, the repressor domain, the zinc-finger domain, the leucine-zipper domain, the LZ-FHK loop, and the forkhead domain. The regulatory regions that are target sites for DNA methylation are indicated by blue and green arrows; and for histone modifications by green arrows. The exon localization of the mutations identified in intrauterine IPEX fetuses or neonates from 11 families is depicted. Annotations refer to coding sequence and protein alteration positions; family ID appears between parenthesis.

IPEX is an inborn error of immunity classically caused by mutations on the X-linked gene *FOXP3*, that codes for a transcriptional factor essential for the maintenance of thymus-derived regulatory T cells, or Treg (18, 19), which suppress autoimmune responses, hence, maintaining homeostasis and tolerance (20). Notwithstanding, several cases of IPEX-like patients with wild-type *FOXP3* have been recently reported, some of them bearing mutations on other immune regulatory genes (21). *FOXP3* has 12 exons, the first exon lying in the 5' untranslated region (19, 22). The protein coded by this gene, FOXP3, is a member of the forkhead/winged-helix family of transcriptional regulators and has five functional domains: a proline-rich amino-terminal repressor domain, followed by the zinc-finger (ZF) domain and leucine zipper (LZ) domains, required for protein-protein interactions, the LZ-FKH loop, and the carboxy-terminal forkhead domain (FHK), which is critical for DNA binding and nuclear localization (19, 23). Figure 2 shows a schematic representation of *FOXP3* exons and the corresponding protein functional domains.

FOXP3 has pre-mRNA alternative splicing and its most common isoform is Δ2 (exon-2 skipped) which lacks the nuclear export signal. CD4^+^CD25^+^Treg cells constitutively express nuclear FOXP3, whereas CD4^+^CD25^−^ T cells transiently express FOXP3 in the cytoplasm upon activation (24). Although the functional role of all FOXP3 isoforms is not yet fully understood, it is interesting that patients with frameshift mutations on *FOXP3* resulting in exon 2 deletion but retaining expression of FOXP3Δ2, have milder forms of IPEX (25). FOXP3 expression is under epigenetic regulation that involves conserved non-coding enhancer sequences (CNS), located in the promoter region, in the first intron (CNS1 and 2) and just after the first coding exon (CNS3), which are target sites for DNA methylation and histone modifications. Progressive CNS2 demethylation occurs along thymic Treg cell development (26). Considering that alternative splicing and epigenetic regulation depend on the integrity of *FOXP3* intronic regions, it explains why some patients with intronic mutations have reduced FOXP3 expression and mild to severe forms of IPEX (14, 27, 28).

In order to fulfill its role as the master driver of Treg lineage commitment (20), *FOXP3* expression is under an intricate mechanism of microRNA (miRNA)-mediated post-transcriptional regulation. MiRNAs are ~22 nucleotide long single strand non-coding RNA molecules that control the robustness of gene expression by targeting mRNA for degradation or translational repression. The first human Treg microRNA signature was obtained by Rouas et al. (29) who compared the miRNA expression profiles of umbilical cord Treg cells X non-Treg cells. These authors found that *FOXP3* expression in Treg cells is negatively regulated by miR-31—that directly binds to the 5′ UTR region of *FOXP3* mRNA—and positively, although indirectly, regulated by miR-21. Subsequently, many studies on the role of miRNAs in Treg differentiation and stability were conducted, mostly using murine models. These studies revealed a more complex picture, with a least 20 miRNAs exerting important effects on Treg development, control of FOXP3 stability and translation, and suppression of Treg function, such as miRNA-155, regulated by *FOXP3* and involved in Treg proliferation, and miR-428, a hominid-specific miRNA, that potently and specifically up-regulate *FOXP3* expression accelerating the differentiation of human naïve cells to induced Tregs (30–32). Additionally, it should be recalled that *FOXP3* interacts with several RNA-binding proteins, thus playing a role in the posttranscriptional mechanisms involved in RNA stability (33). The growing knowledge on miRNAs affecting *FOXP3* expression and Treg functions may pave the way for the discovery of new therapeutic approaches for autoimmunity or other immune-driven diseases (34–36).

### *FOXP3* Mutations and Genotype-Phenotype Correlations in IPEX

In 2008, Gambineri et al. (37) reported the clinical and molecular profile of 14 unrelated affected males with IPEX caused by different mutations on FOXP3, pointing out to the lack of correlation between FOXP3 expression and disease severity. Later, studying a large series of 88 IPEX patients from 66 unrelated families, Gambineri et al. (21) were able to correlate the type of mutation with the patents' phenotype, showing that the complete IPEX triad (enteropathy, skin disease, and endocrinopathy) was, by far, more common in patients bearing in-frame deletions (83%) and frameshift mutations (67%). Still, the severity of symptoms was not correlated with the mutation type. Exon stratification showed the majority of the mutations lying in the functional domains, particularly in the exons coding for the FHK, leucine zipper, and repressor N-terminal domains. These data were confirmed by a recently published study on exon stratification of *FOXP3* mutations (14). Finally, a revision of 70 different *FOXP3* mutations accomplished by Bachetta et al. (23) concluded for a lack of genotype-phenotype correlation in IPEX, although drawing attention to specific mutations, such as c.1189 C > T, associated to fetal or neonatal death in several patients.

### *FOXP3* Mutations in Intrauterine IPEX

Nine different *FOXP3* mutations were found in 21 affected individuals (16 confirmed by nucleotide sequencing) of 11 unrelated families with cases of intrauterine IPEX (Figure 1 and Table 1). Noteworthy, six of these mutations are nonsense or frameshift mutations leading to stop codons and truncated non-functional proteins, spanning from exon 1 to the N-terminal side of exon 9, just at the beginning of the forkhead domain (FKH). The other three are missense mutations on FKH exons 9–11 (Figure 2): (i) c.1033C > T, with predicted deleterious effect on FOXP3 function (11); (ii) c.1087A > G, determining a Ile363Val substitution in the middle of the second α-helix of the FKH domain, where conservation of Ile363 is considered critical for the function of FOXP3 (38), and (iii) c.1189C > T, which leads to a remarkable alteration in the electrostatic potentials of the FHK domain, interfering in the interactions between FOXP3 and its target DNA (39). Therefore, it is reasonable to assume all these mutations either abrogate the expression of FOXP3 or alter its functionality, thus contributing to the severe presentation of intrauterine IPEX.

Regarding their exon stratification, four out of nine mutations are located in the exons coding for the FHK domain. The six mutations causing protein truncation are on exons 1, 3, 6, 7, 8, and in the N-terminal side of exon 9. The deleterious missense mutations lay on exons 9, 10, and 11 in the FHK domain. Disease presentation was severe in all cases. Interestingly, the outcomes in these cases, and even their clinical and pathological data, were fairly correlated with the mutation type and location (Figure 2, Table 1). All patients affected by mutations on exons 1, 8, and in the N-terminal side of exon 9, died *in utero*. Conversely, the mutations on exons 6, 7, 10 were not associated with miscarriages, a condition that, in intrauterine IPEX cases, occurs during the second trimester of gestation, when the fetal immune system becomes operational (40). Only the mutations on exons 3 (family 2) and 11 (families 1 and 9) caused different outcomes: two miscarriages and two births in family 2, one miscarriage and one birth in family 1, and one birth in family 9. It is also worth to mention the reports of four cases of non-intrauterine IPEX patients affected by the mutation present in family 6 (c.749delA, exon 7) who survived through childhood (41–43), and one who reached adult life (44), as well as the case described by Kobayashi et al. (38), where the patient was affected by the same mutation present in family 8 (c.1087A > G, exon 10) and survived to childhood. This phenotypic variability (Figure 1) may be related to the mutation itself and/or to the different genomic backgrounds in which those mutations exerted their effects. In the cases above, where the same mutation in different families results in drastically distinct phenotypes, it seems likely that “modifier” genes or gene combinations in the family “background” mediate either “protective” or “aggravating” effects. These would be interesting to investigate, as they may give indications concerning the genetics of “conventional” autoimmune diseases. A similar picture of variable phenotype severity for the same *FOXP3* mutation in different genetic backgrounds is also emerging from mouse studies (see below).

### Screening Tools for IPEX

The immunocytochemical analysis of FOXP3 expression in peripheral cells or in biopsy samples cannot predict the presence and type of genetic alterations in *FOXP3* (45). Therefore, it is mandatory to perform mutation detection analyses in order to ensure an accurate diagnosis (37). Since there are other monogenic defects leading to IPEX-like syndromes, Gambineri et al. (21) employed a custom sequencing array to target the exons and flanking sequences of panel of 50 genes that may cause inborn errors of immunity. By using this panel, these authors studied a cohort of 173 patients with IPEX phenotype from 143 unrelated families, finding that only 88 (50.9%) patients had mutations in *FOXP3*. From 2010 onwards, the discovery of single-gene inborn errors of immunity heavily relies on next-generation DNA sequencing (NGS) technologies, particularly whole exome sequencing (46, 47), allowing the identification of more than 420 genes associated with inborn errors of immunity (48).

## Other Monogenic Defects Leading to IPEX-Like Syndromes

The revision of the clinical data previously reported for patients with other monogenic Tregopathies—namely those not associated with *FOXP3* mutations but also resulting in regulatory T cell deficiency—found intrauterine onset in four individuals from two unrelated families with mutations of interleukin-2 receptor (IL-2R) β chain (IL2RB) gene (21, 33, 49–51). We were not able to clearly identify *in utero* affected individuals in other IPEX-like syndromes, however. Gambineri et al. (21) compared the mean age of onset of enteropathy in a large group of classic IPEX with a group of IPEX-like patients and found 7.8 and 17.3 months, respectively. Age at the first manifestations in loss-of-function mutations affecting the loci *IL2RA* (CD25), *IL2RB, STAT5B* and *LRBA*, and in *CTLA-4* and *BACH2* haploinsufficiency, and in gain-of-function *STAT-3* mutations will now be briefly considered.

Among the patients affected by defects of IL-2 receptor in α or β chains, the first manifestations occur very early, similarly to the ones observed in *FOXP3* mutations. There are only four cases so far described with CD25 deficiency, i.e., the IL-2 receptor α chain. The affected patients show distinct clinical presentations with features of IPEX-like syndrome but also with high susceptibility to extracellular bacteria, fungal, and viral infections (52–55). All these cases had an early onset of the infectious and/or immunedysregulation manifestations. A case described by Bezrodnik et al. (55) presented severe atopic dermatitis, chronic diarrhea, and severe respiratory infection at the 6th day of life, needing hospitalization until she was 6 months old, but no family information was available. Another female infant had diffuse eczema and severe diarrhea during the 1st month of life (54). Caudy et al. (53) described a male baby, born after an uneventful pregnancy and delivery, who presented severe diarrhea, IDDM, and respiratory symptoms at 6 weeks of age. The first case of CD25 deficiency described by Sharfe et al. (52) had his first manifestations at 8 months of age.

More recently, homozygous mutations of *IL2RB* (IL-2 receptor β chain) were identified in five unrelated families with four different mutations, all resulting in severe early-onset multi-system autoimmunity and high susceptibility to viral infections, particularly CMV and EBV (51, 56). Most of the 10 individuals with available clinical data had their first manifestations in the first semester of life, two term infants presenting diarrhea and failure to thrive at 1 month-old. It is remarkable that in one family, there were a 31-week-gestation premature female neonate, who died 2 h after birth due to respiratory failure, and two stillbirths of 25 and 30 weeks of gestational age, and no other sibling with later-onset disease, thus resembling intrauterine IPEX families (51). All these three individuals were found to have intrauterine growth retardation, reduced fetal movement, and skin desquamation, with hyperkeratosis and significant infiltration of B and T lymphocytes on immunohistochemistry. Additional clinical information or histological data from other tissues were not available. Kindred D in the description by Zhang et al. (51) presented a p.Q96^*^ stop-gain mutation, not previously found in any database, leading to significant protein truncation and no IL-2Rβ expression. In another family also reported by Zhang et al. (51), a 37-week-gestation neonate with severe restriction of intrauterine growth was born with dermatitis, developing severe refractory diarrhea in the 1st days of life. In the pedigree of this last family, there is an IUFD case, without additional information. Therefore, among the five families so far described with *IL2RB* mutations, two had intrauterine affected individuals, two preterm babies, and two stillbirths, all apparently with severe growth restriction (57).

Regarding the STAT5b deficiency, besides short stature due to growth hormone (GH) insensitivity, the clinical features included eczema, chronic diarrhea, lymphoid interstitial pneumonia, cytopenias, and increased susceptibility to infections (49). We reviewed seven cases from five unrelated families, finding out that most of them presented low weight and normal length but no other significant features at birth, the immunologic manifestations appearing during the first decade of life, with some cases presenting skin manifestations in the 1st year of life (58–61). STAT5b is a component of both the IL-2 receptor and GH signaling pathways. Recently, dominant negative heterozygous *STAT5B* mutations were detected in three families with GH insensitivity and immune dysregulation, where the affected individuals presented eczema and high IgE levels, without infections (62). Despite the lack of precise age-of-onset information, there is no indication of very early disease manifestations.

Among the 116 LRBA-deficient patients described in the literature, we found 32 (27.6%) with the first manifestations appearing along the 1st year of life, most of them presenting IPEX-like clinical picture (63–71). There are two patients with neonatal identification of the genetic defect due to affected siblings, but clinical manifestations appeared later. *CTLA-4* haploinsufficiency usually manifests later, being 11 years the mean age of onset, and with only three cases manifesting during the 1st year of life, and none in the neonatal period, exploring the large series of 133 cases recently described by Schwab et al. (72).

There are descriptions of early-onset autoimmune manifestations in patients with gain-of-function (GOF) *STAT3* mutations (73, 74). Among 13 individuals from 10 families with a variety of clinical features—most having lymphadenopathy, polyautoimmunity (cytopenias, enteropathy, IDDM, hypothyroidism, and lymphocytic interstitial pneumonitis), infections, and short stature—only three patients presented the first manifestations before completing the 1st year of age, without any reference to the neonatal period (73).

*BACH2* haploinsufficiency was recently identified in three patients from two unrelated families. All affected individuals had colitis: in one patient, the first intestinal manifestations occurred during infancy, and much later in the other two (75).

## Prenatal Investigation of Intrauterine IPEX

**Figure 3:**
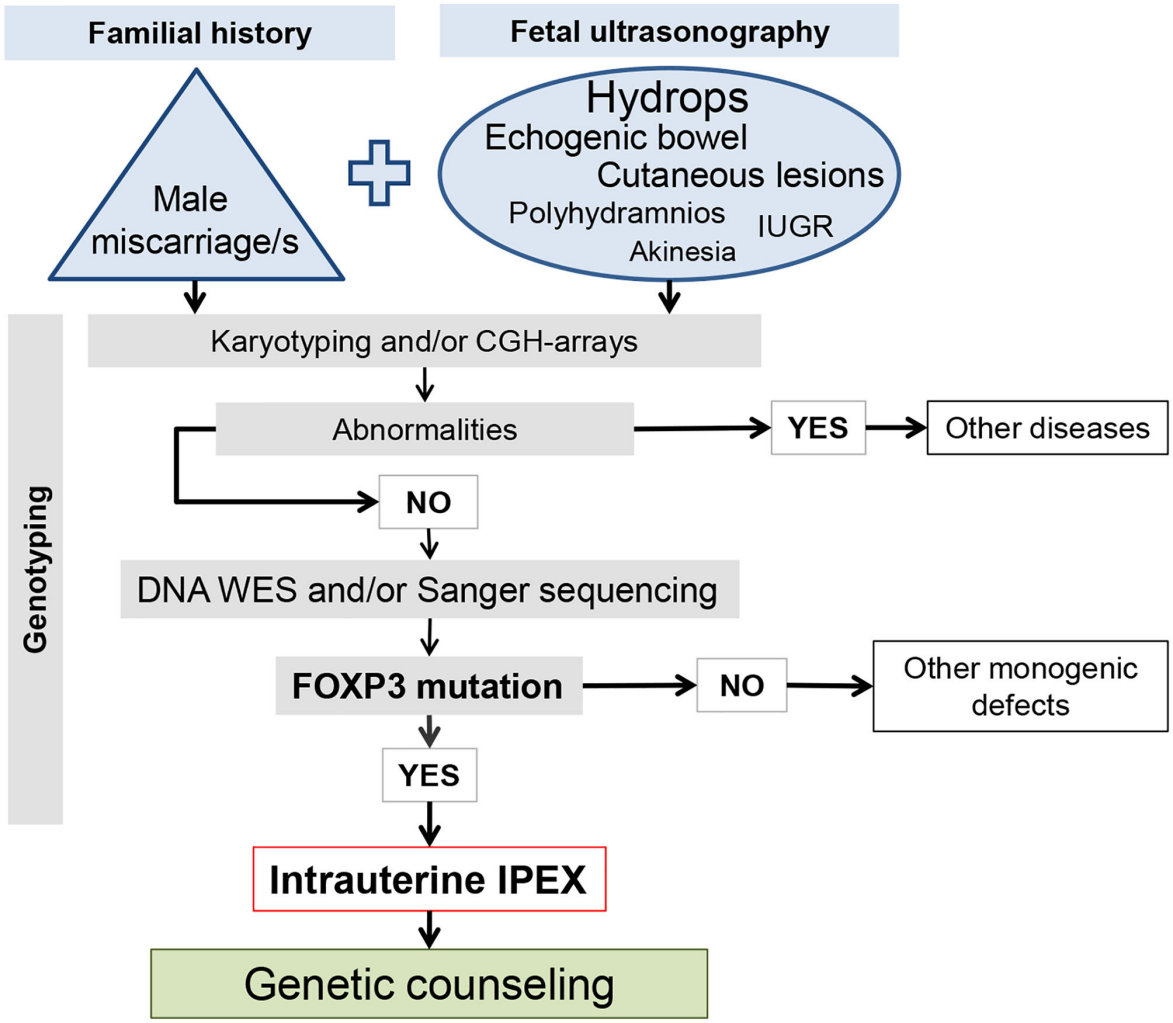
Work-flow diagram for the prenatal identification of intrauterine-onset IPEX. The size of the letters in the ellipse of the ultrasound observation is proportional to the number of the affected cases.

The burden of intrauterine fetal deaths and stillbirths continues to be a major public health problem everywhere, being always necessary to attempt the identification of underlying defects, particularly in cases with familial recurrence (76). It is estimated that 25% of stillbirths are attributable to genetic causes (8, 77). Although IPEX is considered as an extremely rare disease, with no well-established frequency in the population, professionals involved in prenatal care must be attentive to its detection, with the most important warning signs being: (i) recurrent male fetal losses, stillbirths, or premature infants in the family history (see references in Table 1); (ii) dilated bowel loops or hyperechogenic bowel on fetal ultrasonography (US), mainly if associated with skin alterations (6, 7, 10, 12, 16); and (iii) plurivisceral inflammation with eosinophils found in fetal post-mortem pathology (10, 11). Diagnostic confirmation is reached by karyotyping and subsequent DNA sequencing, nowadays by whole exome sequencing (WES), being the mutation always confirmed by Sanger DNA sequencing (8, 10, 47, 48). Regarding bowel abnormalities in fetal US, there are multiple potential etiologies, such as cystic fibrosis, congenital anomalies of gastrointestinal tract, congenital diarrhea disorders, being IPEX an example of the last group (16). Figure 3 depicts a work-flow diagram for the prenatal identification of intrauterine-onset IPEX.

## Severity of Phenotype Variability in *foxp3* Mutant Mice

A spontaneous null mutation in the mouse *Foxp3* gene leads to the scurfy phenotype a fulminant multiorgan autoimmune syndrome resembling human IPEX and affecting only males. The scurfy mutation (Foxp3^sf^) results from a 2-bp frameshift insertion which encodes a truncated gene product lacking the C terminal-forkhead domain (78). The original Foxp3^sf^ mutation has been maintained into the reference strain C57BL/6 in which most immunological studies are conducted. The BALB/c mouse strain is less frequently used in present days, though fully healthy. C57BL/6 and BALB/c mice present dense polymorphisms at many genes and loci. In a paper addressing whether Foxp3 expression in thymic epithelial cells contributes to the scurfy phenotype, Chang et al. (79) incidentally presented survival curves from few animals suggesting anticipated death in Foxp3^sf/y^ BALB/c when compared to Foxp3^sf/y^ C57BL/6. Intrigued by the possibility that different genetic backgrounds could condition the age of death caused by the same severe mutation, we set out to formally test this hypothesis. We designed breeding strategies allowing production of scurfy mice in number large enough for statistical analysis. The purity of the genetic background was guaranteed by repeated inbreeding with C57BL/6 and BALB/c mice (>10 generations) themselves coming from regularly renewed colonies. While the age of death of Foxp3^sf/y^ C57Bl/6 was found to spread from 3 weeks to 4 months of age, with a C50 at 60 days (*N* = 59), Foxp3^sf/y^ BALB/c mice were more homogenous (life span of 10–20 days) and most animals were moribund or dead by 20 days of age (*N* = 41). Finally, F1 animals, heterozygotes for all C57BL/6 and BALB/c allelic variants, phenocopy the more resistant C57BL/6 strain (*N* = 61), indicating genetic dominance.

Other *Foxp3* mutations in mice result from genetic engineering. The knock-out mutation is a large deletion that removes exons 1–5. When reporting this null mutation, Fontenot et al. (80) stated “Male *Foxp3–*mice succumb to an aggressive lymphoproliferative autoimmune syndrome virtually identical to that of the *Foxp3sf* mutant.” The Foxp3^K276X^ mutant, from Lin et al. (81), carries an engineered one nucleotide substitution in exon 8, leading to a premature STOP codon, actually copying a mutation found in human IPEX. This allele was backcross into C57BL/6 and BALB/c mice for eight generations by Haribhai et al. (82), who reported all BALB/c dead by the age of 20 days while C57BL/6 animals succumbed between 30 and 90 days of age.

Together, these data evidenced that the genetic background modulates the severity of phenotype and age at death of two *Foxp3* deleterious mutations. In turn, this finding indicates that allelic variants at yet to be identified genes, or combinations thereof—act as modifiers in scurfy disease, either as an amplifier (BALB/c) and/or possibly as a minifier (C57BL/6).

In comparing mice and humans, it is essential to keep with the developmental time scale. Mice are born immunologically immature when compared to human newborns. Notably, peripheral T cells are sparse or undetectable in new born mice while thymocytes export is effective during the second trimester of human fetus development. It is therefore not surprising that intrauterine scurfy has not been reported in mice. Nevertheless, investigators and caretakers used to follow mouse colonies where the Foxp3^sf^ mutation runs, easily identify the mutation carriers inside a litter by 3–4 days of age.

## Discussion

The study of intrauterine IPEX may give a relevant contribution for elucidating the role of fetal Treg cells. While the critical role of maternal Treg cells in fetal implantation and maternal immune tolerance during pregnancy is well established, the functional role of fetal Treg cells needs deeper understanding (83, 84). Fetal Treg cells are normally detected at the end of the first trimester of pregnancy, and expected to expand in numbers to play their critical role in self-tolerance along the second and third trimesters (84). These cells are believed to suppress fetal anti-maternal immunity as well as fetal immune responses to self-antigens. It is remarkable that the proportion of CD25^+^FOXP3^+^ Treg cells in peripheral fetal lymphoid organs reaches 15–20% of all CD4^+^ T cells, which represent the highest value ever attained during life (83). Healthy newborns exhibit increased Treg cell frequencies and absolute numbers when compared to adults, with an inverse correlation between cell numbers and gestational age (85). Hence, the more premature the neonate is, more elevated is the number of Treg cells in the umbilical cord blood, with most cells presenting a naive phenotype. Otherwise, the fetuses with deleterious *FOXP3* mutations supposedly do not have their natural Treg cells properly developed, and self-tolerance is certainly not fully established, as proposed in the original description of fetal-onset IPEX by Xavier-da-Silva et al. (4). It is likely that the autoimmunity “suppressive” role of Tregs is paramount in development, since emerging repertoires of T and B cells are known to be particularly multi/autoreactive (86, 87), in part due to the absence of TdT expression (88). If, on the one hand, multi/autoreactivity allows for the selection and generation of Tregs (89), it will also endow other T cells with potentially pathogenic autoimmune repertoires that will manifest in the (genetic) absence of Tregs. In order to explain the successful term pregnancies of *FOXP3* mutation carriers, one can postulate that maternal Treg cells, by crossing the placenta or by any other putative mechanism, could play a role in regulating the fetal immune system, therefore delaying autoimmune manifestations to post-natal life (4, 84).

Regarding the organ and tissue targets in intrauterine IPEX, the most frequent observations have been: (i) the pancreas was infiltrated by T-lymphocytes in most fetuses examined as early as 18 weeks of gestational age, (ii) the gut was affected in all fetal and stillbirth cases with available ultrasound data, (iii) cutaneous manifestations were described in five out of 21 individuals with clinical data available (Table 1), and (iv) hemolytic anemia as the possible origin of fetal hydrops and death of most individuals represent the most intriguing feature since its frequency would be high in this particular IPEX group. A recent review of 195 IPEX patients revealed that 191 (97.9%) presented enteropathy, 121 (62.1%) had skin manifestations, 104 (53.3%) manifested endocrinopathies, and in only 75 (38.5%) hematologic abnormalities were detected (14), reinforcing the classic IPEX clinical triad. In other words, the characteristic targets of IPEX polyautoimmunity are similar, while the respective frequencies are quite different, comparing intrauterine-onset cases with the post-natal “conventional” IPEX, for reasons not fully understood yet.

It can be hypothesized that IPEX fetuses in which erythrocytes are early targets of autoimmunity are prone to develop precocious hemolytic anemia. Hence, as a consequence, they would be the ones who evolve to hydrops and intrauterine death. In fact, histopathologic findings of necropsy from IPEX hydrops fetuses are highly suggestive of the presence of hemolysis (4, 6, 10). Since IPEX-associated hemolytic anemia is a typical autoimmune phenomenon, we advance the hypothesis that IPEX fetal hydrops is secondary to an early-onset autoimmune hemolytic anemia, and would also like to propose that IPEX-associated hydrops should be considered as an immune process, instead of being classified as a non-immune fetal hydrops, as it is commonly found in articles approaching this topic (6, 90). Hemolytic anemia in intrauterine IPEX would be an autoimmune condition, differently from alloimmunization caused by Rh incompatibility, mediated by placental transfer of maternal antibodies. Nonetheless, both are mediated by immune mechanisms.

Interestingly, the pedigree analysis of the 11 IPEX families reviewed here revealed intrauterine-onset cases only, without individuals with late-onset IPEX manifestations. A comparison between the two large families studied by Powell et al. (91) and by Shehab et al. (8) shows that the former had 17 postnatally affected males in five generations, and just one stillbirth and one miscarriage, without mention of gender. Inversely, in the family described by Shehab et al. (8), there are 19 IUFD in four generations, without any post-natal cases. Although all the intrauterine IPEX cases bear loss-of-function *FOXP3* mutations, there is no clear mutation-phenotype correlation enabling one to place such severe cases as resulting from a particular mutation type, as summarized in Figure 2. The missense mutation c.1189C > T, identified in three unrelated families with intrauterine IPEX (4, 5, 12), is associated with neonatal or postneonatal deaths in all these families and presumptively with miscarriages in only one (4), with clinical variability among families. The missense mutation c.1087A > G is associated with hydrops fetalis, lethal in utero reactive T cell infiltration, and recurrent male miscarriages in the family reported by Rae et al. (9), but just with late-onset IPEX with milder presentations in the two unrelated cases described by Kobayashi et al. (38).

Mutations leading to severe defects in α or β chains of the IL-2 receptor are associated with early-onset IPEX-like manifestations, with almost all the reported cases presented their initial manifestations during the first semester of life. In addition to *FOXP3* mutations, the Tregopathy due to *IL2RB* defects was the only other one where part of the cases presented a clear intrauterine onset (51, 56). Despite the small number of families and cases described just a year ago, in a well-characterized family (51) with intrauterine manifestations, all the three siblings were severely affected and did not survive. All had severe growth restriction but, differently from classic IPEX, there is no description of hydrops cases, and all the individuals reached the third trimester of gestation. Interestingly, in another family described by the same authors, a 37-week gestation neonate also presented severe growth restriction.

IPEX is considered an extremely rare disease, with no estimated population prevalence, and probably it is underrecognized, particularly the prenatal cases. The awareness of professionals involved in prenatal care is crucial for scrutinizing the mother's personal and familial history, conducting fetal US exploration, and *FOXP3* mutation detection (Figure 3). Although there are no published data on treatment options for IPEX affected fetuses, the clinical common sense recommends pregnancy to be prolonged—aiming at greater maturity and survival conditions after birth—and the mother to be referred to a maternal-fetal medical center. The administration of tacrolimus to the mother would constitute an option to control fetal autoimmunity. This calcineurin inhibitor has been demonstrated as effective for immunosuppression in IPEX patients (3, 12), it crosses placenta (92), and is considered safe to be administered during pregnancy and breast feeding (93, 94).

## Author Contributions

All authors listed have made a substantial, direct and intellectual contribution to the work, and approved it for publication.

## Conflict of Interest

The authors declare that the research was conducted in the absence of any commercial or financial relationships that could be construed as a potential conflict of interest.

## Supplementary Material

Supplementary Table 1 Main clinical, imaging, and histopathologic features found in 21 individuals from 11 unrelated families with intrauterine IPEX.
